# Subchondral stress fracture of femoral head in a healthy adult

**DOI:** 10.4103/0019-5413.67125

**Published:** 2010

**Authors:** Ashish Anand, A RaviRaj, Gautam Kodikal

**Affiliations:** Center for Joint Replacements, Wockhardt Hospitals, Bangalore, India

**Keywords:** Femoral head, stress fracture, subchondral fracture

## Abstract

Subchondral fracture of the femoral head is an uncommon entity and usually occurs as an insufficiency fracture associated with poor bone quality or as a fatigue fracture in young military recruits. This condition should be considered in the differential diagnosis of acute hip pain in young patients along with transient osteoporosis and avascular necrosis of the hip. We report a case of acute onset hip pain in an asymptomatic healthy adult in which the diagnosis was made by magnetic resonance imaging and the patient responded well to conservative treatment.

## INTRODUCTION

Subchondral fracture of the femoral head is an uncommon entity and usually occurs as an insufficiency fracture associated with poor bone quality or as a fatigue fracture in young military recruits.^1^ It is reported in elderly people and in recipients of renal transplants. Visuri^2^ reported stress osteopathy of femoral head in military recruits. Transient osteoporosis of the hip has clinicoradiological findings that mimic those of a subchondral fracture and is usually seen in healthy middle-aged people not involved in sports.^3^ We present a rare case of subchondral fracture of the femoral head in an asymptomatic healthy adult that healed uneventfully.

## CASE REPORT

A 27-year-old healthy male (software professional) presented to out patient department with complaints of pain in the right hip for the past 1 month. Pain was more pronounced in the front of the hip, gradual in onset, and aggravated on walking for long periods. There was no history of trauma, alcohol abuse, steroid use, or any recent increase in physical activity. There was no back pain or any radiation of pain to the knee. There were no other systemic symptoms.

**Figure 1:**
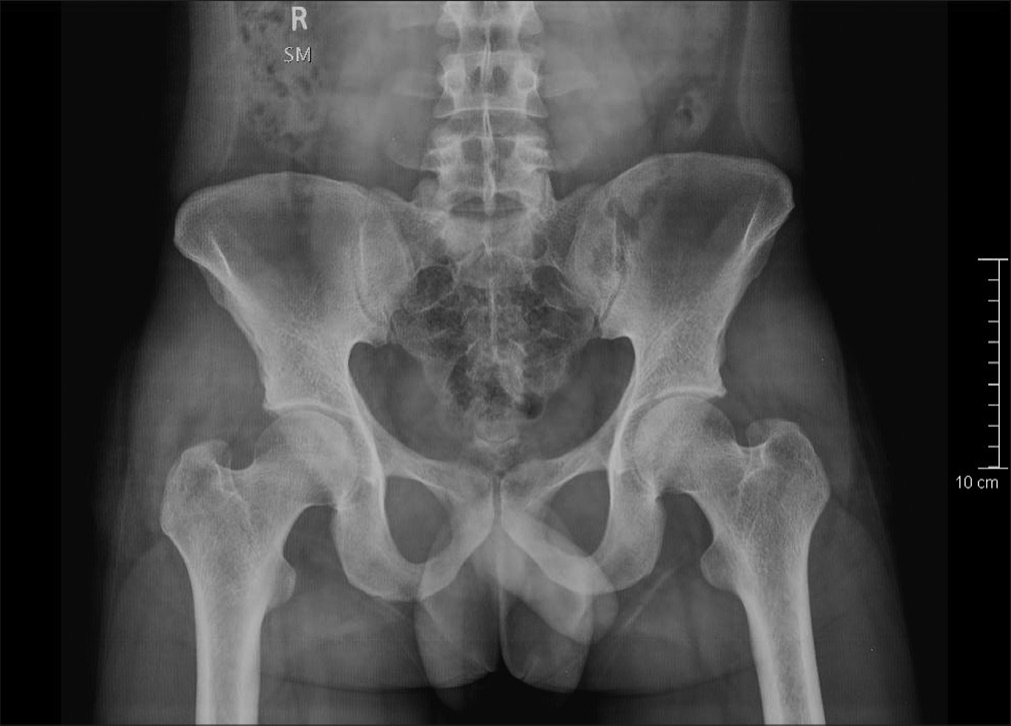
X-ray pelvis with both hip joints anteroposterior view showing no abnormality

**Figure 2:**
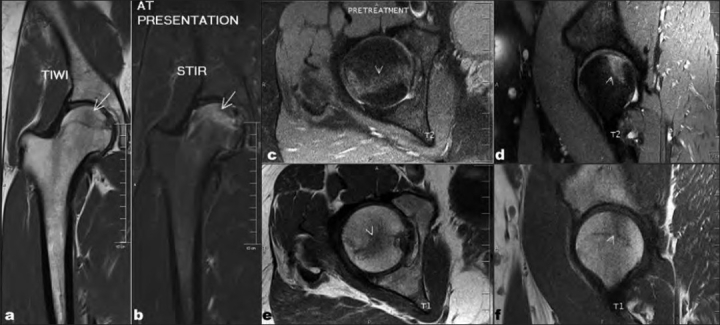
Coronal (a,b) and sagittal (c,d,e,f) magnetic resonance imaging (T-1 and STIR image) showing subchondral marrow edema with a fracture line (marked with arrow and arrow head)

On physical examination, the patient had painful terminal limitation of flexion and internal rotation. There was no limb length discrepancy and the contralateral hip had a normal examination. X-rays of the pelvis with both hips in anteroposterior view [Figure 1] and cross-table lateral view of the right hip revealed no abnormalities. Magnetic resonance imaging (MRI) [Figure 2] of the right hip revealed a fracture line with hypointense signal on both T-1 weighted and T-2 weighted images along with subchondral bone edema. A bone densitometry was carried out, which revealed normal bone density.

The possible differential diagnosis was avascular necrosis, transient osteoporosis, and subchondral fracture. The treatment options were discussed with him and he was offered a trial of conservative treatment with the explanation that he would need a repeat MRI. At this time, the patient was put on non weight bearing crutch walking and was also put on Alendronate 70 mg once in 2 weeks.

**Figure 3:**
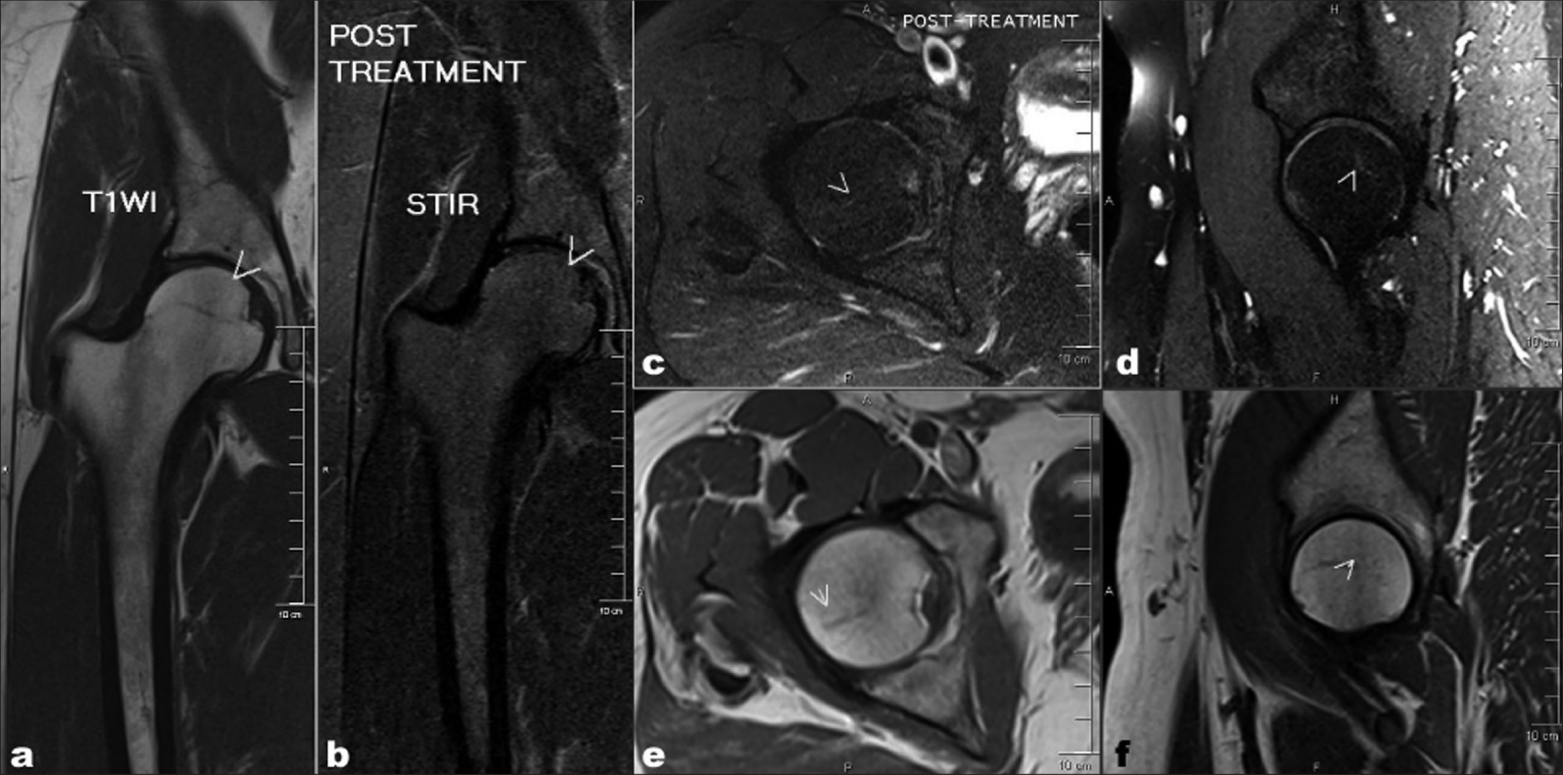
Coronal (a,b) and sagittal (c,d,e,f) magnetic resonance imaging (T-1 and STIR image) performed 8 weeks later showing resolution of bone edema and absence of fracture line (marked with arrowhead)

The patient followed-up at 8 weeks revealed a normal clinical examination and absence of symptoms. There was no pain on flexion and internal rotation at this time. A repeat MRI [Figure 3] performed at 8 weeks showed resolution of bone edema and disappearance of the fracture line.

The patient did not have any symptoms at 1 year follow-up.

## DISCUSSION

The diagnosis in our case was established with MRI, which showed marrow edema along with the fracture line. To the best of our knowledge, there has been only one previous report on healthy adults by Kim *et al*., who reported their observations on five asymptomatic young adults that healed spontaneously with conservative treatment.^4^

Subchondral stress fracture of the femoral head has been attributed to insufficiency fracture associated with poor bone quality 5–8 in the elderly population or as a fatigue fracture in young military recruits.^1^,^2^ When it occurs in young military recruits, it is usually secondary to increased activity levels, with MRI revealing fracture with or without collapse. Patients without collapse heal with restriction of activity while those with collapse eventually need total hip replacement.^1^ In a series of four patients with insufficiency fracture reported by Buttaro *et al*., 8 three of the four patients required total hip replacement because of significant collapse of the head and significant restriction of movement.

MRI in fatigue fracture shows a serpiginous line parallel to the articular surface with a high signal on fat suppression proximal to the fracture line. The two important differential diagnoses are osteonecrosis and transient osteopenia of the hip. MRI in all the three conditions reveals bone marrow edema.

Osteonecrosis is usually seen in men above the age of 40 with a history of excessive alcohol intake or chronic steroid administration.^10^ In osteonecrosis, MRI reveals well-defined focal lesion with a low-intensity band concave to the articular surface with the absence of a high-signal proximal to the band on fat-suppressed view. MRI in cases of osteonecrosis shows a subchondral collapse.^9^

Transient osteoporosis of the hip on clinicoradiological findings mimic that of subchondral fracture albeit the fracture line, but it usually occurs in healthy middle-aged people not involved in sports or similar activities. X-rays do not reveal focal bone loss or subchondral collapse.^11^ MRI shows a diffuse and a homogeneous decreased signal intensity on T-1 weighted images and increased signal intensity on T-2 weighted images.^5^

To summarize, subchondral stress fractures of the femoral head in healthy adults are uncommon and in the absence of femoral head collapse they have a good prognosis. The advances in MRI technology allow the clinician to pick up this uncommon entity and also follow it up to see if it responds to conservative treatment.
